# A Comparison of Immersive vs. Non-Immersive Virtual Reality Exercises for the Upper Limb: A Functional Near-Infrared Spectroscopy Pilot Study with Healthy Participants

**DOI:** 10.3390/jcm12185781

**Published:** 2023-09-05

**Authors:** Milos Dordevic, Olga Maile, Anustup Das, Sumit Kundu, Carolin Haun, Bernhard Baier, Notger G. Müller

**Affiliations:** 1 Department of Chronic and Degenerative Diseases, Faculty of Health Sciences (FGW), Potsdam University, 14476 Potsdam, Germany; 2 Department of Neurology, Otto-von-Guericke University, 39120 Magdeburg, Germany; 3 Faculty of Informatics, Otto-von-Guericke University, 39106 Magdeburg, Germany; 4 Edith-Stein Fachklinik, 76887 Bad Bergzabern, Germany; 5 University Medical Center of the Johannes Gutenberg University Mainz, 55131 Mainz, Germany

**Keywords:** virtual reality, fNIRS, upper limb, immersive, hemoglobin

## Abstract

Functional near-infrared spectroscopy (fNIRS) allows for a reliable assessment of oxygenated blood flow in relevant brain regions. Recent advancements in immersive virtual reality (VR)-based technology have generated many new possibilities for its application, such as in stroke rehabilitation. In this study, we asked whether there is a difference in oxygenated hemoglobin (HbO_2_) within brain motor areas during hand/arm movements between immersive and non-immersive VR settings. Ten healthy young participants (24.3 ± 3.7, three females) were tested using a specially developed VR paradigm, called “bus riding”, whereby participants used their hand to steer a moving bus. Both immersive and non-immersive conditions stimulated brain regions controlling hand movements, namely motor cortex, but no significant differences in HbO_2_ could be found between the two conditions in any of the relevant brain regions. These results are to be interpreted with caution, as only ten participants were included in the study.

## 1. Introduction

Virtual reality (VR)-based platforms are used nowadays in many settings [1], including the restoration of upper limb function. This application is particularly common in the research on rehabilitation of stroke survivors, whose upper limb movement is impaired in about half of all patients and, therefore, requires intensive practice usually supervised by a physiotherapist. Here, several studies reported beneficial effects of additional practice in VR [2,3], related to both functional outcomes [4] and neuroplasticity [5] in stroke patients.

A wide variety of VR systems are available on the market—from those expensive and customizable to the low-cost ones, using either immersive or non-immersive VR technology, sensor- or marker-based, with or without controllers. In recent years, immersive VR systems—usually containing a head-mounted display (HMD)—have been developed and produced at ever increasing pace. One of the obvious advantages is the optional three-dimensional (3D) environment, which offers a more realistic experience to users. Prior to the advent of technological advancements enabling 3D systems, VR settings predominantly consisted of 2D environments, usually via computer/laptop screens or projectors, known as non-immersive VR. Since the emergence of the 3D technology, many studies have sought to examine disparities between them (for a review, see [6]). However, others reported that in 3D systems, users become more engaged in the environment, being active participants, rather than just being passive observers, which brings the possibility of explorative interaction with the environment [7], but also better memory performance [8] and perception [9]. In addition, immersive designs in 3D can produce greater psycho-physiological responses in healthy users [10] as well as greater improvements in recovery of stroke patients [11], compared to non-immersive ones. Nevertheless, it remains unknown if physiological signatures, as observed by functional near-infrared spectroscopy (fNIRS), differ between the two modalities (3D vs. 2D)—which may be of great relevance for the recovery of stroke survivors.

Functional near-infrared spectroscopy (fNIRS) is suitable for the investigation of the effects of movement on cerebral oxygenation and hemodynamics—it is able to quantify changes in the concentration of oxygenated hemoglobin (oxyHb) and deoxygenated hemoglobin (deoxyHb) non-invasively [12]. A number of previous studies used fNIRS to assess various upper limb movements and conditions, such as simple hand movements [13], hand-grip strength [14], skill development [15], focal upper limb dystonia [16], stroke rehabilitation [3,17], cortical reorganization [18] and so on. However, to the best of our knowledge, up to now there are no studies which have combined VR and fNIRS to track upper limb movements. Moreover, no study so far has compared upper limb movements in 2D and 3D using fNIRS.

Therefore, for this study, we developed a VR-based paradigm to evaluate the functionality of the upper limb. Our primary inquiry revolves around the potential dissimilarities in activation, as observed by fNIRS, within the corresponding brain regions between:

(a) The immersive VR and the non-immersive desktop (nVR) conditions;
(b) Both of these conditions compared to the baseline (B) condition.

Considering the findings of previous studies, we hypothesized increased activation in relevant brain areas—primarily motor cortex and neighboring areas—in both VR and nVR conditions compared to B. Regarding possible differences between the immersive (VR) and non-immersive (nVR) conditions, based on the existing literature, no clear hypothesis could be formulated.

## 2. Materials and Methods

### 2.1. Study Design and Participants

This study was organized as cross-sectional, with one group and two conditions (immersive and non-immersive), in accordance with the declaration of Helsinki. Ten healthy young participants (24.3 ± 3.7 years old, three females) were included in this proof-of-concept study.

### 2.2. VR Setup

All measurements took place in a quiet testing room with closed and blinded windows with the lights off, in order to reduce any possible sound and light interference.

**Figure 1:**
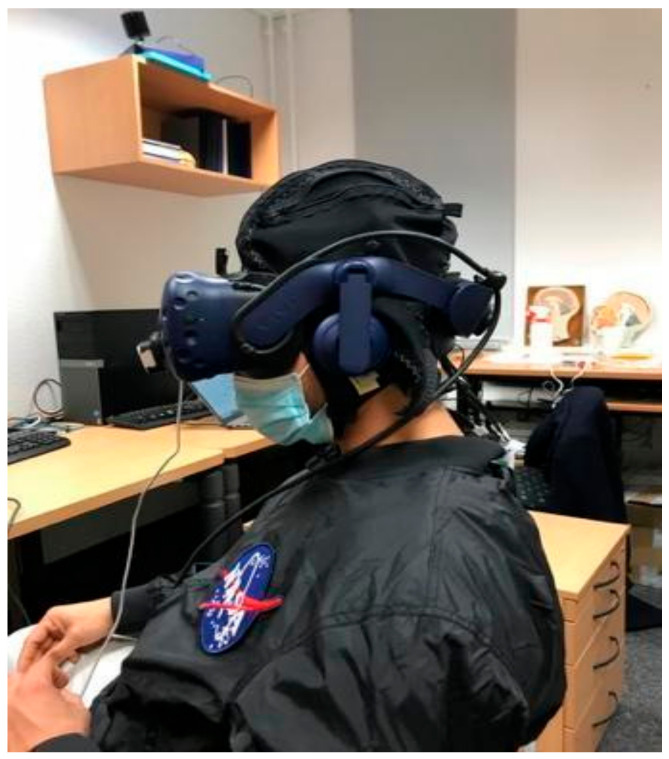
Participant wearing HTC-Vive and fNIRS (covered with a dark cap).

First, the whole procedure was explained to the participant, followed by one familiarization trial with each paradigm, both in immersive (HMD) and non-immersive settings (on the laptop screen). Next, the participant was asked to sit in the chair in front of the table on which the laptop (Dell, Round Rock, Texas, US) with screen size of 61 cm, full HD resolution) was placed at a distance of about one meter from the participant’s head. Then, the complete fNIRS system (see below for detailed description) was mounted on the participant’s head and tested for signal quality. Following this, the participant was instructed to don the HMD (HTC-Vive Pro, Taoyuan City, Taiwan) on their head while ensuring optimal adjustments for utmost comfort. On the front side of the HMD, the Leap Motion (Leap Motion Company, San Francisco, CA, USA) controller was placed so that hand movements could be captured within the virtual environment. Once the entire setup was ready, the paradigms were played one after the other, in the same order for each participant. They were informed verbally when to begin and stop performing the task. Upon completion of paradigms in the immersive condition, the HMD was removed from participant’s head and the Leap Motion controller placed on the table facing upwards. This setup allowed for the continued detection and projection of hand movements onto the laptop screen. With these preparations complete, the testing of paradigms in the non-immersive condition commenced (Figure 1).

### 2.3. VR Paradigm—Bus Riding

**Figure 2:**
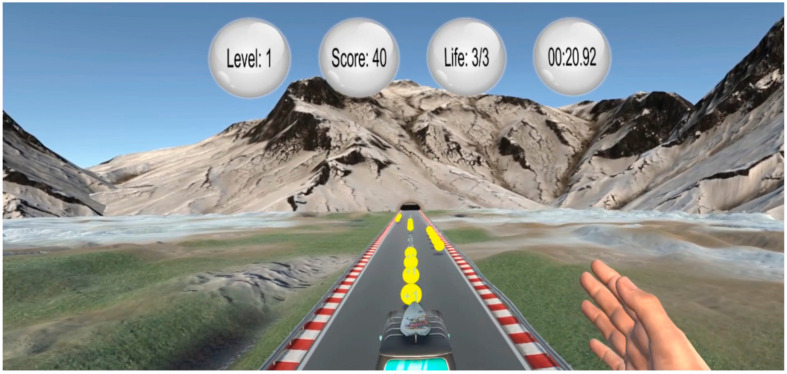
VR-based bus riding paradigm.

A specially developed paradigm for this study, called bus riding, was used in order to stimulate hand movement. In this paradigm, participants used their hand to steer a moving bus, as shown in Figure 2. By moving the right hand, be it the wrist or fingers, in the metacarpophalangeal joints, right and left, the bus responsively adjusts its position while transitioning between lanes. The participant’s hand remains perpendicular to the lane and hand movements are in palmar- and dorsi-flexion. In addition, participants should avoid obstacles and collect coins placed on the road. The more the game progresses, the higher the driving speed, and thus the difficulty level, becomes. Steering the bus completely off the road leads to re-initiation of the game and return to the starting position. During each run, the goal was to collect a maximum number of points.

Participants were allowed to play in both immersive and non-immersive settings for three runs per 30 s, with 30 s of passive break between runs, whereby the hand was placed on the table. Additional baseline recordings were taken immediately before the first and after the last run, in both settings.

### 2.4. Data Acquisition

Hemodynamic brain signals were obtained using a NIRSport 2 system (NIRx Medical Technologies, Glen Head, NY, USA) in accordance with the protocol used by a previous study [19]. The system was equipped with seven detectors and eight light source optodes emitting at 760 and 850 nm. The electrodes were separated by an inter-optode distance of 30 mm and covered sensorimotor brain areas such as premotor and supplementary motor cortex (PMC–SMA), primary motor cortex (M1), somatosensory association cortex (SAC), and inferior parietal cortex (IPC) on both hemispheres.

Cortical hemodynamic changes were recorded at a frequency of 10.17 Hz in 22 channels. The fNIRS optodes were placed according to the 10–20 EEG system [20] on top of an fNIRS cap.

### 2.5. fNIRS Preprocessing and Analysis

Hemodynamic time series were analyzed using Satori (Brain Innovation BV, Maastricht, The Netherlands). Raw wavelength output from the fNIRS system was trimmed by 10 s at the beginning and 10 s at the end of each run, and then converted into optical densities through application of the modified Beer–Lambert law [21]. Motion artefacts were removed through temporal derivative distribution repair (TDDR) [22] and monotonic spike removal. Other artefacts (breathing, Mayer waves, heartbeat, low-frequency drifts) were addressed through linear detrending, low-pass filtering (Butterworth, 0.4 Hz) and high-pass filtering (Butterworth, 0.01 Hz) [23]. Finally, the optical densities were converted into concentration data and normalized into z-scores.

The task related brain activation between immersive (VR) and non-immersive (nVR) was analyzed with a GLM-based short channel regression, using the one of the eight short-separation channels that was closest to the long-separation channel. As the fNIRS signal is contaminated by the back-reflections of the skin and the skull, it was proposed to use these short source-detector separation optodes as regressors, as they are sensitive to the blood flow in the superficial layers of the human brain [24]. This should improve the signal across long separation fNIRS channels, as regional blood flow can differ substantially across the entire PFC. To control for multiple comparisons during channel wise *t*-tests, the false discovery rate (FDR) correction (*q* < 0.05) [25] was applied. The resulting *t*-map was displayed with Satori and used for further interpretation of the results. For all tests, a *p*-value of <0.05 was considered significant.

## 3. Results

Complete datasets for all three conditions (VR, nVR, B) from ten healthy young participants were obtained and analyzed in this study (age 24.3 ± 3.7, all right-handed, three females, all university students).

### 3.1. Immersive (VR) vs. Baseline (B)

**Figure 3:**
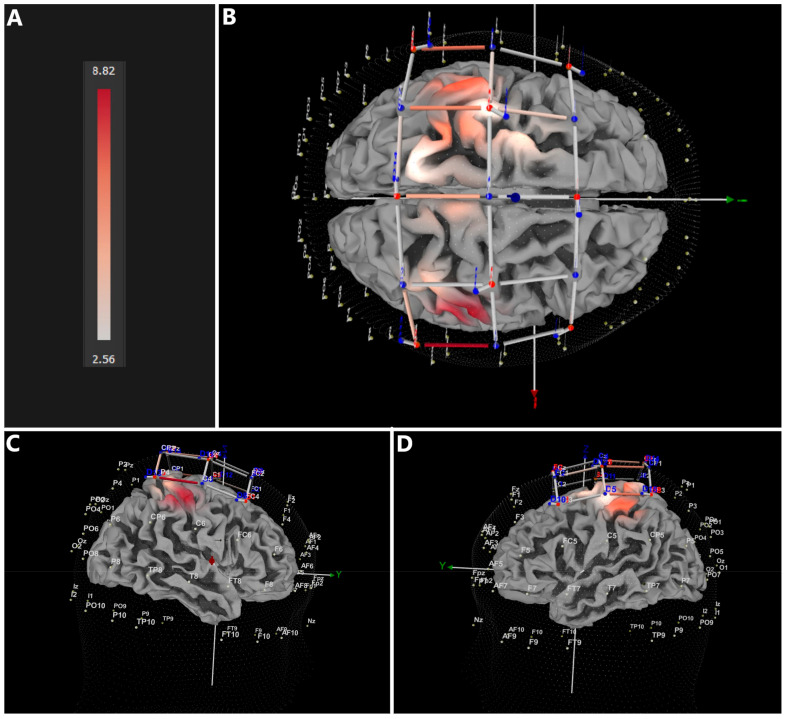
Brain regions showing significant FDR-corrected differences between immersive VR and baseline conditions; (**A**) CI heatmap, (**B**) top view, (**C**) lateral right view, (**D**) lateral left view.

As depicted in Figure 3 and listed in Table 1, the VR condition (VR) induced significantly more increase (CI = 3.755 − 8.820) in oxy-hemoglobin (HbO_2_) compared to the baseline condition (B). The most affected brain regions corresponded with primary motor cortex (M1), but also premotor and supplementary motor cortices. Moreover, the most activated topographical location in M1 corresponded well with regions responsible for processing arm- and hand-related information.

### 3.2. Non-Immersive (nVR) vs. Baseline (B)

**Figure 4:**
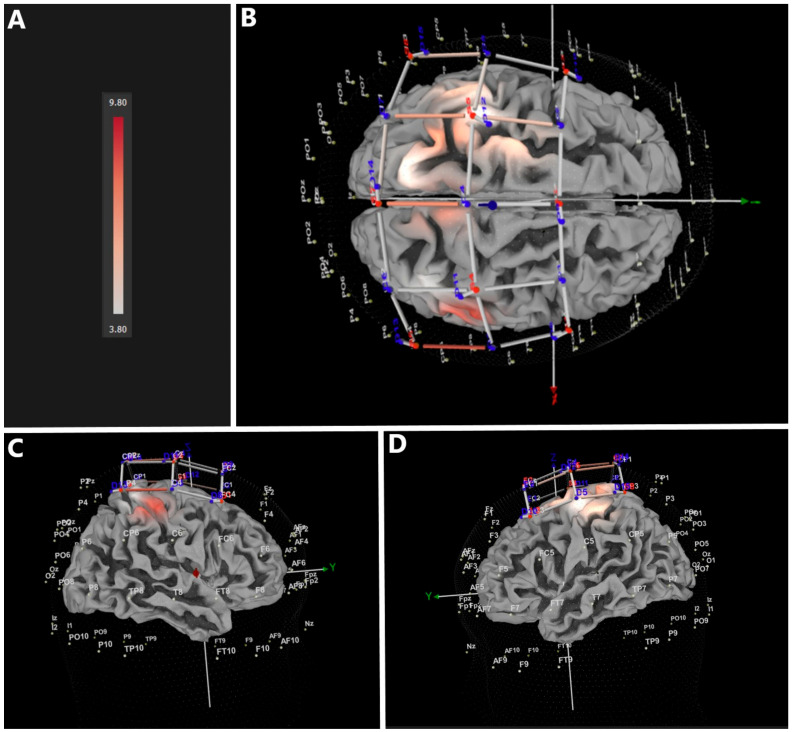
Brain regions showing significant FDR-corrected differences between non-immersive (nVR) and baseline conditions; (**A**) CI heatmap, (**B**) top view, (**C**) lateral right view, (**D**) lateral left view.

Similar to the immersive VR condition, participants revealed significantly larger increase (FDR-corrected) in HbO_2_ in the non-immersive (nVR) compared to the B condition (CI = 3.802–9.796) (Figure 4 and Table 1). The affected regions corresponded to those found in the immersive VR condition.

### 3.3. Immersive (VR) vs. Non-Immersive (nVR)

**Figure 5:**
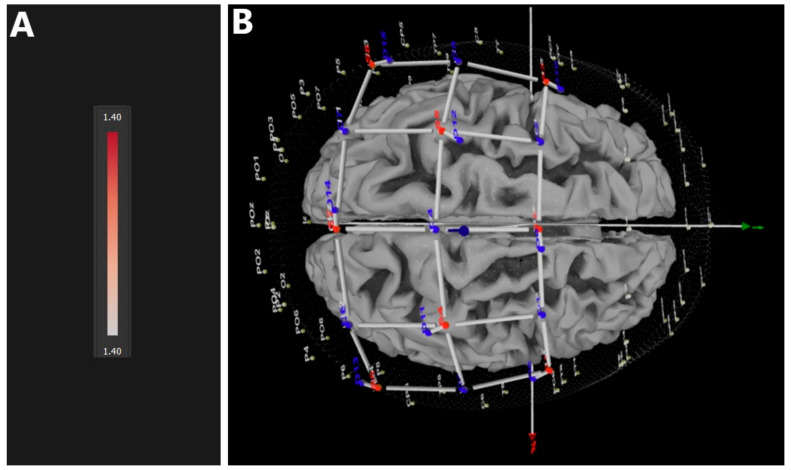
FDR-corrected brain map showing no differences between immersive (VR) and non-immersive (nVR) conditions. (**A**) CI heatmap, (**B**) top view.

In contrast to comparisons of both VR and nVR conditions with baseline, no significant difference in HbO_2_ could be found between immersive VR and non-immersive (nVR) conditions in any of the relevant brain areas (Figure 5). No behavioral differences between the two conditions were found.

## 4. Discussion

This study confirmed the hypothesized effects of both immersive VR and non-immersive (nVR) conditions of a specially developed paradigm for inducing activation in brain regions controlling hand movements, namely increased HbO_2_ in the motor cortex and neighboring brain regions relative to the baseline (B). No significant differences could be found between VR and nVR conditions in HbO_2_ in any of the relevant brain regions. Thus, this study was the first one to determine such effects for all three conditions (VR, nVR, B).

In accordance with earlier studies, we found that fNIRS can be used to detect an increase in HbO_2_ in corresponding brain regions as a result of upper limb movement. Previous studies used fNIRS for various applications related to upper limb movement—including various single hand tasks [13], movement intention recognition for brain–computer interfaces [26,27,28], performance of spatial tasks using upper limbs [29], etc. Moreover, numerous studies are available on fNIRS application in stroke patients suffering from upper limb paresis—such as in assessment of unilateral vs. bilateral upper limb training [30], determination of effects of active and passive upper limb training modes [3], assessment of sensory-motor cortex reorganization following upper limb paresis due to stroke [18], and so on. However, none of these studies combined fNIRS and specially developed paradigms in VR to investigate changes in HbO_2_ resulting from upper limb movement.

Brain regions in which an increase in HbO_2_ was detected in this study are in agreement with the literature. Hand and wrist motion leads to an increase in HbO_2_ in the motor cortex and neighboring regions in the contralateral hemisphere [31,32]. A bilateral hemispheric increase in HbO_2_ in response to right upper limb movement is already a known phenomenon, especially when motor imagery is involved [33]. Movements in the wrist joint in certain directions can also elicit bilateral hemispheric responses; in addition, wrist joint movements in left and right directions cause increases in HbO_2_ in more brain regions than upward and downward movements [32]. In addition, the VR paradigm might have led to the activation of the observation network of the brain, which differs from the pure movement network [34].

VR-based applications have gained a significant momentum in recent years—rather than being passive observers, users are allowed to engage in various VR environments as active participants, which has been exploited by gaming, sports, medicine, education, military, and also research [7]. It was reported that user satisfaction is generally higher in VR compared to other methodologies, which might lead to higher learning rates and skill improvement [7]. An earlier study reported no difference between VR and desktop conditions in memory performance of younger adults, unlike in older adults, who performed better in the desktop condition [35]. A recent review reported that immersive VR can be considered beneficial for rehabilitation of stroke patients suffering from upper limb paresis [36]. Other studies support the efficacy of fully immersive VR over non-immersive VR for improving 3D perception and evaluation of cognitive functions [8,9], but also for enhancing the sense of presence and emotional response [10]. A recent meta-analysis on stroke patients reported beneficial effects of both immersive and non-immersive VR-based rehabilitation protocols, with immersive using head-mounted displays (HMDs) inducing the largest improvements in upper extremity function, as assessed by the Fugl-Meyer test [6]. Using EEG, it was found that learning in immersive VR leads to better knowledge transfer compared to conventional learning [37]. Despite these behavioral differences in some previous studies, we could not find brain activity differences between the VR and nVR condition in the present study. Although we cannot exclude the possibility that with a larger sample and with other methods for assessing brain activity (e.g., fMRI), differences between VR and nVR could have been observed, the present null finding speaks against large differences in brain activity induced by VR as opposed to nVR conditions. Note that no previous study has assessed changes in blood oxygenation (HbO_2_), as measured by fNIRS, resulting from upper limb movement in VR and nVR conditions—this precludes the direct comparability of our results. Additionally, it prompts the need for future studies using a larger sample of participants, to confirm or contradict our findings. Nevertheless, the results of our study provide novel insights into this important topic, which can be of relevance not only in health but also in various clinical conditions, such as stroke.

Despite providing significant results, our study contains some limitations. This particularly relates to the sample size, since only ten participants were included. However, considering the proof-of-concept nature of this study and its novelty, the idea was to take care of the resources and still assess the feasibility, including the specially designed paradigm coupled with the latest generation state-of-the-art fNIRS system. Even with the small number of participants, we obtained significant results when comparing both active conditions (VR and nVR) to the baseline condition; still, we could not detect any significant differences between the two active conditions, which might be—at least partially—due to a low number of participants. Thus, additional studies on larger samples of participants are needed. Moreover, we could not find any behavioral differences between the two active conditions (VR and nVR)—perhaps an assessment of elderly participants using this paradigm would lead to behavioral differences as well. Additionally, although we applied the 10–20 standardized system, participants’ heads vary in size and shape, therefore we cannot determine the exact neuroanatomical hemodynamic localization—therefore, future studies with more accurate methodologies are required.

In conclusion, results of this study brought two important findings: (I) compared to baseline, upper limb movements in both VR and nVR conditions lead to a significant increase in HbO_2_ in corresponding motor cortex and neighboring brain regions, and (II) no significant difference in HbO_2_ between the VR and nVR conditions could be found. These results are to be interpreted with caution, as only ten participants were included and fNIRS signal could not be obtained from very precise locations. Nevertheless, these results can be considered beneficial for all future studies using both immersive and non-immersive VR-based technology, both in healthy participants and various patient groups suffering from brain damage.

## Figures and Tables

**Table 1:** Confidence intervals (CI) of the difference for comparisons showing significant differences (VR vs. B and nVR vs. B) in oxy-hemoglobin (HbO_2_).

Comparison	Confidence Interval (CI) of the Difference in HbO_2_	
	Min	Max
VR vs. B	2.560	8.820
nVR vs. B	3.802	9.796

## Data Availability

All data are stored at the FGW—Potsdam University and can only be obtained under special permission.
